# Association between admission anemia and long-term mortality in patients with acute myocardial infarction: results from the MONICA/KORA myocardial infarction registry

**DOI:** 10.1186/s12872-018-0785-5

**Published:** 2018-03-09

**Authors:** Miriam Giovanna Colombo, Inge Kirchberger, Ute Amann, Margit Heier, Christian Thilo, Bernhard Kuch, Annette Peters, Christa Meisinger

**Affiliations:** 1 MONICA/KORA Myocardial Infarction Registry, Central Hospital of Augsburg, Augsburg, Germany; 2 Institute of Epidemiology, Helmholtz Zentrum München, German Research Center for Environmental Health (GmbH), Neuherberg, Germany; 3 Chair of Epidemiology, Ludwig-Maximilians-Universität München, UNIKA-T, Augsburg, Germany; 4 Department of Internal Medicine I – Cardiology, Central Hospital of Augsburg, Augsburg, Germany; 5 Department of Internal Medicine/Cardiology, Hospital of Nördlingen, Nördlingen, Germany

**Keywords:** Myocardial infarction, Anemia, Hemoglobin, Mortality

## Abstract

**Background:**

Previous studies have shown that the presence of anemia is associated with increased short- and long-term outcomes in patients with acute myocardial infarction (AMI). This study aims at examining the impact of admission anemia on long-term, all-cause mortality following AMI in patients recruited from a population-based registry. Contrary to most prior studies, we distinguished between patients with mild and moderate to severe anemia.

**Methods:**

This prospective study was conducted in 2011 patients consecutively hospitalized for AMI that occurred between January 2005 and December 2008. Patients who survived more than 28 days after AMI were followed up until December 2011. Hemoglobin (Hb) concentration was measured at hospital admission and classified according to the World Health Organization (WHO). Mild anemia was defined as Hb concentration of 11 to < 12 g/dL in women and 11 to < 13 g/dL in men; moderate to severe anemia as Hb concentration of < 11 g/dL. Adjusted Cox regression models were calculated to compare survival in patients with and without anemia.

**Results:**

Mild anemia and moderate to severe anemia was found in 183 (9.1%) and 100 (5%) patients, respectively. All-cause mortality after a median follow-up time of 4.2 years was 11.9%. The Cox regression analysis showed significantly increased mortality risks in both patients with mild (HR 1.74, 95% CI 1.23–2.45) and moderate to severe anemia (HR 2.05, 95% CI 1.37–3.05) compared to patients without anemia.

**Conclusion:**

This study shows that anemia adversely affects long-term survival following AMI. However, further studies are needed to confirm that anemia can solely explain worse long-term outcomes after AMI.

**Electronic supplementary material:**

The online version of this article (10.1186/s12872-018-0785-5) contains supplementary material, which is available to authorized users.

## Background

Anemia found in patients with acute myocardial infarction (AMI) and measured at hospital admission has been identified as an independent predictor of adverse outcomes such as cardiac events, major bleeding as well as short- and long-term mortality [1–5]. Defined according to the World Health Organization (WHO), anemia is present in women and men if hemoglobin (Hb) concentration falls below 12 g/dL and 13 g/dL, respectively [6]. Compared with the prevalence in the general population (3.8%) [7], anemia is more frequently encountered in patients hospitalized for cardiac events [8, 9]. Ranging from 11% to 38% the presence of anemia varied widely across prior studies in patients with AMI [10, 11].

The majority of previous studies in patients with AMI focused on comparing those with and without anemia neglecting severity of anemia. However, since it is a common condition found in hospitalized patients, severity of anemia might be important to consider [11]. In addition, results from long-term studies covering observation periods beyond 5 years are scarce. Therefore, the aim of this study was to examine the association between admission anemia and long-term, all-cause mortality in patients with AMI recruited from the MONICA/KORA myocardial infarction registry and to incorporate severity of anemia into the analysis.

## Methods

The data for this study were derived from the Myocardial Infarction Registry that was established in Augsburg as part of the WHO project MONICA (Monitoring Trends and Determinants in Cardiovascular disease) in 1984. All coronary deaths and cases of non-fatal AMI occurring among the inhabitants of the city of Augsburg and the 2 adjacent counties (600,000 inhabitants) have been continuously registered since then. The population-based registry was included into the KORA (Cooperative Health Research in the Region of Augsburg) framework when the MONICA project ended in 1995.

Patients aged between 25 and 74 years, who were admitted to one out of 8 hospitals in the study area were included. Written informed consent had to be obtained before patients were included into the cohort. More detailed information on case identification, diagnostic classification of events and quality control of the data can be found in previous publications [12, 13]. Trained study nurses interviewed the participants during hospital stay using a standardized questionnaire. In order to confirm the information provided by the patients and to collect additional information, the patients’ medical chart was reviewed. Both methods of data collection and questionnaires have been approved by the ethics committee of the Bavarian Medical Association (Bayerische Landesärztekammer) and the study was performed in accordance with the Declaration of Helsinki.

We conducted this prospective study in consecutive patients hospitalized for AMI between January 1, 2005 and December 31, 2008. Patients were followed up for all-cause mortality, the outcome of this study, until December 31, 2011. The vital status of study participants after hospital discharge was determined through population registries located in- and outside the study region. Patients were included in this study if they survived longer than 28 days after AMI had occurred. Those with missing information on both admission Hb concentration (*n* = 68) as well as relevant covariates (*n* = 176) were excluded. The final study population consisted of 2011 patients with AMI.

Presence of anemia was defined based on Hb concentration (g/dL) measured at hospital admission and patients were categorized according to WHO classification of anemia [6]. Mild anemia was defined as Hb concentration of 11 to < 12 g/dL in women and 11 to < 13 g/dL in men. Moderate to severe anemia was present when Hb concentration was below 11 g/dL. Since only thirteen patients had severe anemia (Hb < 8 g/dL), no further subdivisions were made.

In order to examine whether an impaired renal function was present, we used the estimated glomerular filtration rate (eGFR) and applied the Modification of Diet in Renal Disease (MDRD) study equation (eGFR (ml/min/1.73 m^2^) = 186.3 × (serum creatinine^− 1.154^) x (age^− 0.203^) × 0.742 (if female) × 1.212 (if black)) [14] to calculate it. Risk factors such as history of angina pectoris, prior myocardial infarction, hypertension, hyperlipidemia, diabetes mellitus, stroke as well as patients’ smoking habits were covered during the interview conducted by the study nurses and confirmed by chart review (except for history of stroke and smoking habits). Body mass index (BMI; kg/m^2^), systolic and diastolic blood pressure as well as heart rate and AMI classification (ST-segment elevation myocardial infarction (STEMI), non-ST-segment elevation myocardial infarction (NSTEMI) or bundle branch block) were derived from chart review only. Echocardiography, ventriculography and radionuclide ventriculography were used to determine whether patients had a reduced left ventricular ejection fraction (LVEF < 30%). Furthermore, medications administered at discharge were documented. The majority of patients received antiplatelet agents, angiotensin-converting enzyme inhibitors (ACEI) or angiotensin-receptor-blockers (ARB), beta-blockers and statins at discharge and therefore, we included these medications as one covariate (4 evidence-based medications (EBM); yes/no). In-hospital procedures such as percutaneous coronary intervention (PCI) and coronary artery bypass graft (CABG) were determined by chart review. Since in-hospital complications rarely occurred, a single covariate was generated including the occurrence of cardiac arrest, pulmonary edema, bradycardia, re-infarction, ventricular tachycardia, ventricular fibrillation or cardiogenic shock.

Possible differences in survival were tested using Kaplan-Meyer plots as well as log-rank tests. Hazard ratios (HR) for all-cause mortality according to anemia status were calculated using Cox regression models. Three different models were calculated: 1) an unadjusted model, 2) a model adjusted for age and sex, and 3) a model adjusted for age, sex, previous MI, angina pectoris, hyperlipidemia, diabetes, stroke, eGFR, heart rate, AMI type, LVEF, discharge medications, PCI and in-hospital complications. Covariates made it into the latter model if the corresponding log-rank-test was statistically significant (*p* < 0.05) and if they proved to make a statistically significant contribution to predicting all-cause mortality in a model together with anemia status. We graphically tested whether the assumption of proportional hazards (parallel lines of log (−log (event)) versus log of event times) was valid for each covariate. Time-dependent interaction terms were included if the assumption was rejected. The covariates age and sex were included into each model independent of statistical significance. Due to frequently missing data, LVEF was entered into the regression model as a dummy coded variable (LVEF < 30%; yes/no/missing). The variance inflation factor (VIF) was used to detect multicollinearity among covariates [15]. Furthermore, we calculated adjusted Cox regression models for increasing observation periods ranging from one to 6 years.

As a sensitivity analysis, we calculated a Cox regression model including all patients who were originally excluded from our study population due to missing information on any covariate (*n* = 176). We adjusted this model for sex and age. Patients without anemia served as the reference category for all analyses. *P*-values of < 0.05 were considered statistically significant. The analyses were performed using statistical software package SAS version 9.2 (SAS Institute Inc., Cary, NC).

## Results

In total, 283 AMI patients (14.1%) were considered anemic based on admission Hb concentration. Of those patients, 183 (64.7%) were mildly anemic, whereas 100 (35.3%) had moderate to severe anemia. Male patients accounted for 75.6% of the total study population and the mean age was 60.9 ± 9.6 years. Further patient characteristics are summarized in Table 1.

**Table 1:** Baseline characteristics and long-term mortality of patients with AMI by anemia status (*n* = 2011)

	Anemia^a^ (*n* = 283)			Non-anemia^d^ (*n* = 1728)	*p* Value
	Total	Mild anemia^b^ (*n* = 183)	Moderate to severe anemia^c^ (*n* = 100)		
Socio-demographic characteristics					
Age (years)	64.8 ± 8.5	64.5 ± 8.5	65.4 ± 8.4	60.1 ± 9.6	< 0.0001
Female	70 (24.7)	43 (23.5)	27 (27.0)	440 (25.5)	0.7838
Living alone, (*n* = 1939)	54 (20.7)	35 (20.2)	19 (21.6)	291 (17.34)	0.4057
Risk factors and medical history					
BMI (kg/m^2^), (*n* = 1934)	27.2 ± 4.8	27.4 ± 4.8	26.8 ± 4.9	28.0 ± 4.5	0.0272
Smoking status, (*n* = 1886)					< 0.0001
Smoker	55 (22.7)	36 (22.5)	19 (23.2)	661 (40.2)	
Ex-smoker	102 (41.2)	70 (43.8)	32 (39.0)	515 (31.3)	
Never smoker	85 (35.1)	54 (33.8)	31 (37.8)	468 (28.5)	
Prior myocardial infarction	41 (14.5)	24 (13.2)	17 (17.0)	165 (9.6)	0.0228
Angina pectoris	71 (25.1)	51 (27.9)	20 (20.0)	282 (16.3)	0.0004
Hypertension	230 (81.3)	142 (77.6)	88 (88.0)	1367 (79.1)	0.0830
Hyperlipidemia	164 (58.0)	107 (58.5)	57 (57.0)	1118 (64.7)	0.0884
Diabetes	123 (43.5)	68 (37.2)	55 (55.0)	488 (28.2)	< 0.0001
Stroke	38 (13.4)	21 (11.5)	17 (17.0)	85 (4.9)	< 0.0001
Laboratory markers					
Hemoglobin (g/dL)	11.2 ± 1.6	12.1 ± 0.6	9.5 ± 1.3	14.8 ± 1.3	< 0.0001
eGFR (ml/min/1.73m^2^)	63.9 (43.4–85.5)	65.9 (47.4–83.4)	60.3 (35.9–88.4)	78.3 (64.4–92.3)	< 0.0001
eGFR < 60 ml/min/1.73m^2^	122 (43.1)	73 (39.9)	49 (49.0)	329 (19.0)	< 0.0001
Clinical characteristics					
Systolic blood pressure (mmHg)	120 ± 17	120 ± 16	121 ± 19	118 ± 15	0.0746
Diastolic blood pressure (mmHg)	68 ± 10	68 ± 10	67 ± 11	69 ± 10	0.1236
Heart rate (bpm)	73 ± 11	73 ± 11	73 ± 10	71 ± 10	0.0004
AMI type					< 0.0001
STEMI	63 (22.3)	46 (25.14)	17 (17.0)	616 (35.6)	
NSTEMI	194 (68.6)	120 (65.6)	74 (74.0)	1019 (59.0)	
Bundle branch block	26 (9.2)	17 (9.3)	9 (9.0)	93 (5.4)	
LVEF (*n* = 1188)					0.0021
LVEF < 30%	4 (2.9)	3 (1.6)	1 (1.0)	38 (2.2)	
LVEF ≥30%	132 (46.6)	87 (47.5)	45 (45.0)	1014 (58.7)	
Missing	147 (51.9)	93 (50.8)	54 (54.0)	676 (39.1)	
Medication at discharge					
Antiplatelet agents	263 (92.9)	172 (94.0)	91 (91.0)	1686 (97.6)	< 0.0001
ACEIs/ARBs	225 (79.5)	149 (81.4)	76 (76.0)	1493 (86.4)	0.0045
Beta-blocker	271 (95.8)	175 (95.6)	96 (96.0)	1728 (96.1)	0.9595
Statins	239 (84.5)	161 (88.0)	78 (78.0)	1636 (94.7)	< 0.0001
4 EBM	178 (62.9)	125 (68.3)	53 (53.0)	1358 (78.6)	< 0.0001
Calcium channel blocker	49 (17.3)	33 (18.0)	16 (16.0)	216 (12.5)	0.0758
Diuretics	184(65.0)	115 (62.8)	69 (69.0)	877 (50.8)	< 0.0001
Insulin	57 (20.1)	30 (16.4)	27 (27.0)	147 (8.5)	< 0.0001
Other antidiabetic agents	51 (18.0)	31 (16.9)	20 (20.0)	225 (13.0)	0.0594
In-hospital treatment					
PCI	139 (49.1)	102 (55.7)	37 (37.0)	1335 (77.3)	< 0.0001
CABG	66 (23.3)	39 (21.3)	27 (27.0)	235 (13.6)	< 0.0001
Any in-hospital complication^e^	51 (18.0)	31 (16.9)	20 (20.0)	229 (13.3)	0.0773
Outcome					
All-cause mortality	85 (30.0)	48 (26.2)	37 (37.0)	156 (9.0)	< 0.0001

*AMI* acute myocardial infarction, *ACEI* angiotensin-converting enzyme inhibitor, *ARB* angiotensin-receptor blocker, *BMI* body mass index, *CABG* coronary artery bypass graft, *EBM* evidence-based medications (antiplatelet agents, ACEIs/ARBs, beta-blockers, statins), *eGFR* estimated glomerular filtration rate, *LVEF* left ventricular ejection fraction, *PCI* percutaneous coronary intervention

Data are presented as n (%), mean ± standard deviation or median (interquartile range (25%-quartile – 75%-quartile))

^a^ Anemia: Hemoglobin (Hb) concentration of < 12 g/dL in women, Hb concentration of < 13 g/dL in men

^b^ Mild anemia: Hb concentration of 11 g/dL to < 12 g/dL in women, Hb concentration of 11 g/dL to < 13 g/dL in men

^c^ Moderate to severe anemia: Hb concentration of < 11 g/dL in men and women

^d^ Non-anemia: Hb concentration of ≥12 g/dL in women, Hb concentration of ≥13 g/dL in men

^e^ Any in-hospital complication includes at least one of the following: cardiac arrest, pulmonary edema, bradycardia, re-infarction, ventricular tachycardia, ventricular fibrillation, cardiogenic shock

Patients without anemia differed from the group with anemia concerning a majority of patient characteristics: they were significantly younger, had a higher BMI and were more likely to smoke (see Table 1). In terms of known comorbidities and other risk factors, the non-anemia group was overall healthier. They were less likely to have diabetes and to have suffered from prior myocardial infarction, angina pectoris and stroke. Additionally, they had a significantly higher eGFR on admission. Patients with anemia were less likely to receive antiplatelet agents, ACEIs/ARBs and statins, but more often received diuretics and insulin at hospital discharge. Patients with anemia more often had a LVEF < 30% and information on LVEF was more frequently missing than in patients without anemia.

**Fig. 1:**
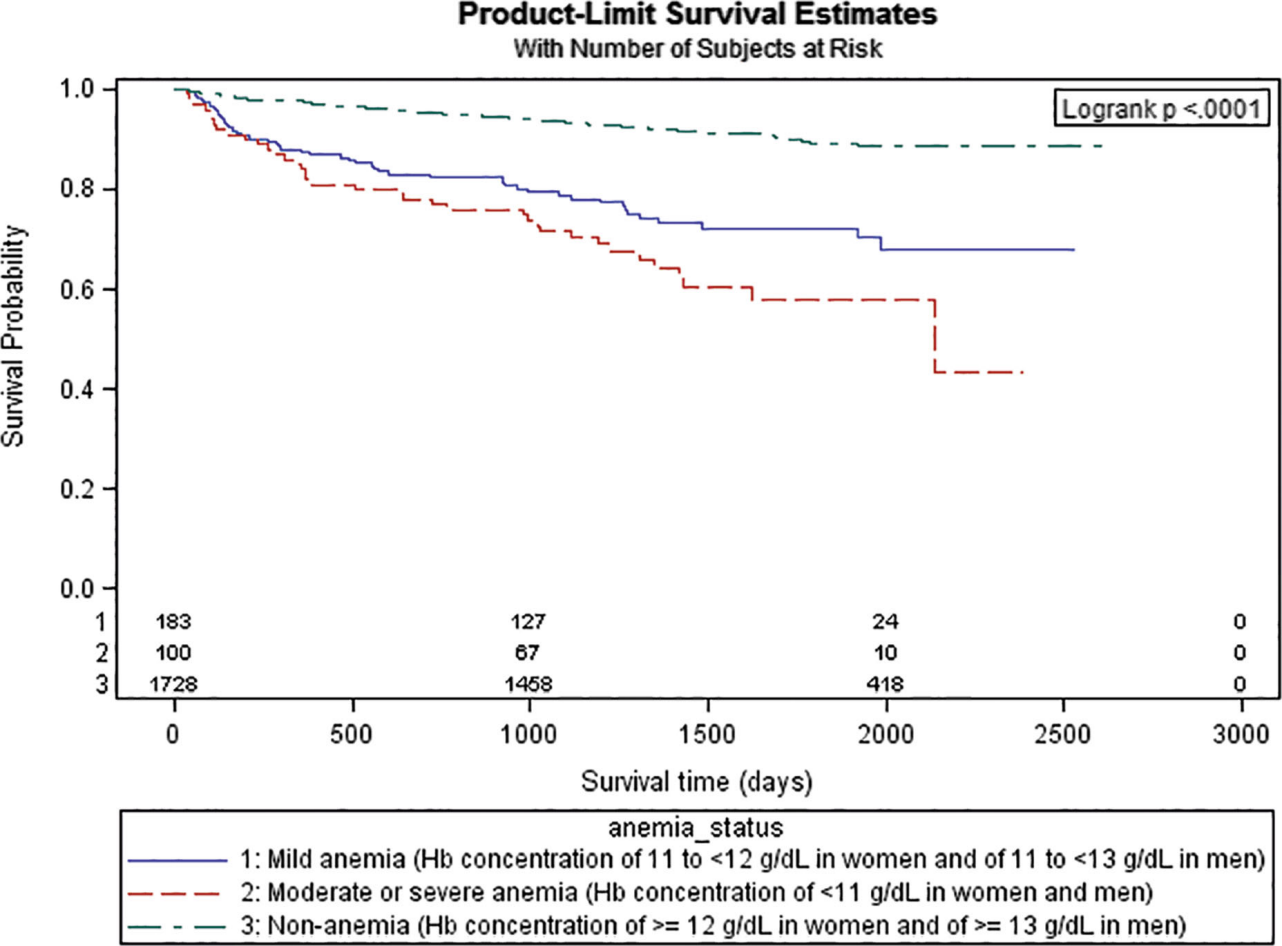
Kaplan-Meier curve with log-rank test *p*-value of 6-year survival by anemia status. Hb, Hemoglobin

During a median follow-up time of 4.2 years (IQR 3.1–5.4), 241 (12.0%) patients with AMI died. Patients with anemia had a significantly higher long-term mortality (*n* = 85, 30.0%) compared to patients without anemia (*n* = 156, 9.0%). A higher percentage of patients died in the group with moderate to severe anemia (*n* = 37, 37.0%) than in the group with mild anemia (*n* = 48, 26.2%). Kaplan-Meier plots showing survival curves stratified by anemia status and the corresponding log-rank *p*-value are provided in Fig. 1. Patients who died during follow-up were significantly older, had a lower eGFR and were more likely to have an impaired LVEF compared to those without an event during follow-up (data not shown). Furthermore, they received four EBM at discharge significantly less often (data not shown).

Results of the Cox regression analyses are shown in Table 2.

**Table 2:** Cox regression models for long-term mortality following AMI by anemia status (*n* = 2011)

	Anemia^a^ (*n* = 283)						Non-anemia^d^ (*n* = 1728)
	Total		Mild anemia^b^ (*n* = 183)		Moderate to severe anemia^c^ (*n* = 100)		
	HR [95% CI]	*p* Value	HR [95% CI]	*p* Value	HR [95% CI]	*p* Value	HR [95% CI]
Unadjusted model	3.99 [3.07–5.20]	< 0.0001	3.35 [2.45–4.68]	<.00001	5.22 [3.64–7.48]	< 0.0001	Ref.
Model 1^e^	3.13 [2.39–4.11]	< 0.0001	2.71 [1.95–3.77]	< 0.0001	3.94 [3.73–5.68]	< 0.0001	Ref.
Model 2^f^	1.85 [1.37–2.49]	< 0.0001	1.74 [1.23–2.45]	0.0017	2.05 [1.37–3.05]	0.0004	Ref.

*AMI* acute myocardial infarction, *CI* confidence interval, *HR* hazard ratio, *Ref* reference category

^a^ Anemia: Hemoglobin (Hb) concentration of < 12 g/dL in women, Hb concentration of < 13 g/dL in men

^b^ Mild anemia: Hb concentration of 11 g/dL to < 12 g/dL in women, Hb concentration of 11 g/dL to < 13 g/dL in men

^c^ Moderate to severe anemia: Hb concentration of < 11 g/dL in men and women

^d^ Non-anemia: Hb concentration of ≥12 g/dL in women, Hb concentration of ≥13 g/dL in men

^e^ Model 1: Adjusted for age and sex

^f^ Model 2: Model 1 + previous myocardial infarction, angina pectoris, hyperlipidemia, diabetes, stroke, eGFR, heart rate, AMI type (STEMI, NSTEMI, Bundle branch block), left-ventricular ejection fraction (LVEF) < 30%, medications at discharge (evidence-based medications (antiplatelet agents, angiotensin-converting-enzyme inhibitor (ACEI), angiotensin-receptor blocker (ARB), beta-blocker, statins), calcium channel blockers, diuretics), percutaneous coronary intervention (PCI) and any in-hospital complication

In the unadjusted model, patients with mild anemia and patients with moderate to severe anemia had significantly increased mortality risks compared to the non-anemia group by factor of 3.35 and 5.22, respectively. With increasing adjustment, HRs decreased but still remained statistically significant. Interaction terms were each included in a regression model together with anemia status due to a rejected proportionality assumption for sex, age, BMI, smoking habits, history of angina pectoris, history of diabetes, AMI type, LVEF, eGFR and heart rate. None of the interaction terms made a statistically significant contribution to the models. Despite the adjustment, patients with moderate to severe anemia still had a 2 times higher mortality risk (HR 2.05, 95% CI 1.37–3.05) compared to the reference group. In patients with mild anemia, the risk of dying was increased by 74% in the final model (HR 1.74, 95% CI 1.23–2.45). Possible multicollinearity among covariates was rejected since the VIF did not exceed the threshold value of 2.5.

Cox regression models for increasing observation periods showed decreasing HRs in both groups with anemia (see Additional file 1: Figure S1). After 1 year, both anemia groups had a 2.4-times increased risk of dying. The risk decreased to HRs of 1.7 and 2.1 in patients with mild anemia and moderate to severe anemia 6 years after AMI, respectively. Estimates drifted apart starting at 3 years of observation period.

The sensitivity analysis showed increased HRs in patients with moderate to severe anemia (HR 4.19 vs. 3.94) and attenuated HRs in patients with mild anemia (HR 2.46 vs. 2.71) compared to the results from our actual study population (see Additional file 2: Table S1). The estimates remained statistically significant.

## Discussion

In the present analysis, we demonstrated that anemia on admission both the mild and moderate to severe type was associated with higher long-term all-cause mortality in patients hospitalized for AMI. HRs attenuated after multivariate adjustment, but a considerable and statistically significant difference in mortality risk persisted. Similar risks for patients in both anemia groups were found 1 year after AMI before they decreased and drifted apart with increasing observation periods.

In patients with coronary artery disease, the prevalence of anemia on admission varied widely across previous studies and ranged from 11% [10] to up to 38% [11]. Compared to most previous studies, the prevalence of anemia in our population (14.1%) was low [1, 8, 10, 11, 16–21]. Those studies focused either only on patients with STEMI or included all patients with acute coronary syndrome (ACS), which might explain the disparities. A higher prevalence of anemia was found in previous studies in patients with AMI [1, 5, 11]. This could derive from the fact that we excluded patients who survived for 28 days or less, which reduced the prevalence of anemia in our study population.

In line, studies with observation periods of at least 1 year found significant associations between admission anemia and long-term mortality [1, 16, 18, 22–24]. Anemia predicted 1-year survival in ACS patients [18] and 2-year survival or AMI in men with ACS [22]. In patients with STEMI, anemia was significantly associated with an increased cardiovascular mortality risk after 21 months [23], major cardiovascular events (MACE) after 5-years [16] and all-cause mortality after 6-years of follow-up [24]. Ducrocq et al. examined 3541 patients with AMI and found a 5-year mortality risk increased by 40% in patients with anemia (HR 1.4, 95% CI 1.2–1.6) [1]. In comparison, the mortality risk found in patients with anemia from our study population was increased by 80%. Among other known risk factors, Ducrocq et al. adjusted their regression analysis for in-hospital bleeding and transfusion [1]. Studies have shown that patients with anemia are more susceptible to experience major bleeding after cardiac events and revascularization [3, 25], which might also affect their long-term mortality risk [1, 25]. Apart from advising to use certain antiplatelet agents with care to avoid bleeding in patients with anemia after AMI [26], current clinical practice guidelines do not provide specific recommendations for the management of anemia in those patients [27]. In terms of AMI treatment using PCI, a study showed that radial instead of femoral access might reduce the risk of bleeding in patients with AMI [28]. Due to the lower risk of bleeding, the radial access might also be preferable when performing PCI in patients with anemia. Furthermore, assuming that patients with anemia are more likely to receive blood transfusion than patients without anemia, an increased risk of “transfusion-associated mortality” [29] might exist. Clinical practice guidelines regarding blood transfusion recommend a Hb threshold of 7–8 g/dL in hospitalized patients [30]. However, transfusion should be taken into consideration in patients with acute coronary syndrome and a Hb concentration of 8–10 g/dL [30]. Data on both in-hospital bleeding and transfusion were not collected in the framework of the registry and, therefore, the possibility exists that we overestimated the mortality risk in patients with anemia. Given the prognostic importance, future studies should include data on bleeding as well as blood transfusions and a Hb threshold for blood transfusion in patients with AMI should be determined.

Furthermore, we subdivided patients with anemia and found increased mortality risks already in patients with mildly reduced Hb concentration. This could have been concealed in previous studies only distinguishing between patients with anemia and those without. Younge et al. examined patients with ACS (defined as STEMI or NSTEMI) who were followed up for over 20 years and found significantly increased mortality risks in those with moderate (HR 1.13) and severe anemia (HR 1.39), but not in those with mild anemia [11]. In their study, patients with anemia were subdivided by tertiles, which deviated from the WHO classification. The cut-off points, especially those for men with mild anemia (12.2–13.0 g/dL vs. 11–13 g/dL in our study) might be responsible for different survival estimates found in their study [11]. Nonetheless, both our and their results stress the need to account for severity of anemia in future studies. Furthermore, our analysis of increasing observation periods showed that severity of anemia might not be important in the first 2 years after AMI but might become more relevant in subsequent years.

Inconsistent with our results, a study in patients with STEMI treated with primary PCI did not confirm an association after 3 years of follow-up [19]. Besides it being a single center study, differences in study population could explain the inconsistency with our results. Furthermore, the authors argue that not anemia itself might negatively impact long-term survival, but rather other comorbidities could explain the worse prognosis [19]. In our study and most previous studies [1, 10, 11, 23], patients with anemia were more likely to be older, were affected by more comorbidities, had an impaired eGFR as well as a lower LVEF. Even though important comorbidities were included in our analysis, data on other measures of overall health status were not available. Additionally, patients with anemia differed from those without anemia regarding in-hospital treatment. They were less often treated with PCI, but more frequently with CABG, which might be an indicator of more advanced coronary artery disease. In line, previous studies demonstrated that anemic patients were less often treated with PCI [18, 21] and experienced worse outcomes after PCI, e.g. increased risks for stent thrombosis, long-term mortality, MACE and bleeding [3, 4, 23, 31]. Furthermore, patients with anemia were less likely to receive 4 EBM at hospital discharge, which is considered the standard of care in patients after AMI and has been shown to significantly reduce long-term mortality [32]. Out of the 4 EBM, both patients with mild and moderate to severe anemia were less likely to receive antiplatelet agents, ACEIs/ARBs and statins compared to patients without anemia. In line, a study in STEMI patients showed that those with anemia were less frequently treated according to guidelines in terms of pharmacological treatment compared to those without anemia [20]. However, less often receiving 4 EBM could also be a consequence of other pre-existing diseases apart from anemia such as impaired renal function [33].

Multiple factors might influence long-term mortality after AMI in patients with anemia. When anemia is present, the amount of oxygen delivered to the heart during AMI is further decreased, myocardial tissue oxygenation is likely to be insufficient and cardiac output is increased [31, 34]. Possibly entailing an impaired recovery after AMI [16], anemia might affect mortality, but cannot solely explain the significantly worse long-term outcomes in patients with AMI. Even though both mild and moderate to severe anemia did predict an increased risk for long-term mortality independent of a number of confounders in our study population, treatment strategies that aim at increasing Hb concentration in patients with AMI and anemia might not significantly benefit long-term survival. In line, a recent randomized controlled trial demonstrated that administering erythropoietin after PCI, a hypoxia-induced hormone that also regulates Hb concentration, did not have beneficial effects on long-term outcomes [35].

Our study is characterized by several strengths. Data was collected in the framework of a population-based registry and patients with AMI were consecutively enrolled. Important risk factors such as comorbidities, in-hospital treatment and complications, relevant laboratory values as well as medications received at hospital discharge were included in our analysis. A longer follow-up than most previous studies and the analysis of increasing observation periods add valuable information to existing research.

This study has limitations. First, even though several risk factors potentially affecting survival after AMI were included, data on cancer, gastro-intestinal or other chronic diseases was not collected. Second, we had no information on the etiology of anemia and how it was treated (e.g. using iron therapy). Knowing the cause of abnormal Hb concentrations would considerably contribute to the understanding of the association between anemia and long-term mortality. Third, any other events occurring after hospital-discharge apart from all-cause mortality and possibly affecting survival could not be monitored. Fourth, a reduced LVEF is a marker for heart failure and data on LVEF was not available in all patients in our study population. Since we included those patients with missing values for left-ventricular ejection fraction we cannot rule out potential bias. Finally, due to the methodological limitations of an observational study, a causal relationship between admission anemia and long-term mortality cannot be established with absolute certainty and the possibility of reverse causation exists.

## Conclusion

Both mild and moderate to severe anemia were associated with significantly increased long-term, all-cause mortality risks in our study population and low admission Hb concentration needs to be considered as a risk factor in patients with AMI. However, even though our results confirm what most other studies have found in patients with AMI before, it remains unclear if anemia alone can predict long-term mortality after AMI or if it is merely a proxy for worse overall health. Future studies need to take severity of anemia, bleeding events and blood transfusion as well as overall health status into account.

## Additional files

Additional file 1: **Figure S1.** Hazard ratios for long-term mortality in patients with AMI and anemia covering increasing observation periods. Reference: Non-anemia: Hemoglobin (Hb) concentration of ≥12 g/dL in women, Hb concentration of ≥13 g/dL in men. Mild anemia: Hb concentration of 11 g/dL to < 12 g/dL in women, Hb concentration of 11 g/dL to < 13 g/dL in men. Moderate to severe anemia: Moderate to severe anemia: Hb concentration of < 11 g/dL in women and men. 95% Confidence intervals (CI) are represented by vertical lines above and below the HR estimates; 95% CI for mild anemia: dashed line; 95% CI for moderate to severe anemia: continuous line. AMI, Acute myocardial infarction; CI, Confidence interval; Hb, Hemoglobin; HR, Hazard ratio. (PDF 49 kb)

Additional file 2: **Table S1.** Results of the sensitivity analysis (*n* = 2187). (DOCX 16 kb)

## Abbreviations

ACEI: Angiotensin-converting enzyme inhibitor
ACS: Acute coronary disease
AMI: Acute myocardial infarction
ARB: Angiotensin-receptor blockers
BMI: Body mass index
CABG: Coronary artery bypass graft
EBM: Evidence-based medication
eGFR: Estimated glomerular filtration rate
Hb: Hemoglobin
HR: Hazard ratio
IQR: Interquartile range
KORA: Cooperative Health Research in the Region of Augsburg
LVEF: Left ventricular ejection fraction
MACE: Major cardiovascular event
MDRD: Modification of Diet in Renal Disease
MI: Myocardial infarction
MONICA: Monitoring Trends and Determinants in Cardiovascular disease
NSTEMI: Non-ST-segment elevation myocardial infarction
PCI: Percutaneous coronary intervention
SD: Standard deviation
SPC: Serum potassium concentration
STEMI: ST-elevation myocardial infarction
VIF: Variance inflation factor
WHO: World Health Organization
