# Supplementary material for: Association between admission anemia and long-term mortality in patients with acute myocardial infarction: results from the MONICA/KORA myocardial infarction registry
Source: BMC Cardiovasc Disord. 2018 Mar 9;18:50. doi: 10.1186/s12872-018-0785-5 (PMC5845173; doi:10.1186/s12872-018-0785-5)
Supplement: Supplementary file 1 — Figure S1. Hazard ratios for long-term mortality in patients with AMI and anemia covering increasing observation periods. Reference: Non-anemia: Hemoglobin (Hb) concentration of ≥12 g/dL in women, Hb concentration of ≥13 g/dL in men. Mild anemia: Hb concentration of 11 g/dL to < 12 g/dL in women, Hb concentration of 11 g/dL to < 13 g/dL in men. Moderate to severe anemia: Moderate to severe anemia: Hb concentration of < 11 g/dL in women and men. 95% Confidence intervals (CI) are represented by vertical lines above and below the HR estimates; 95% CI for mild anemia: dashed line; 95% CI for moderate to severe anemia: continuous line. AMI, Acute myocardial infarction; CI, Confidence interval; Hb, Hemoglobin; HR, Hazard ratio. (PDF 49 kb) [file 12872_2018_785_MOESM1_ESM.pdf]

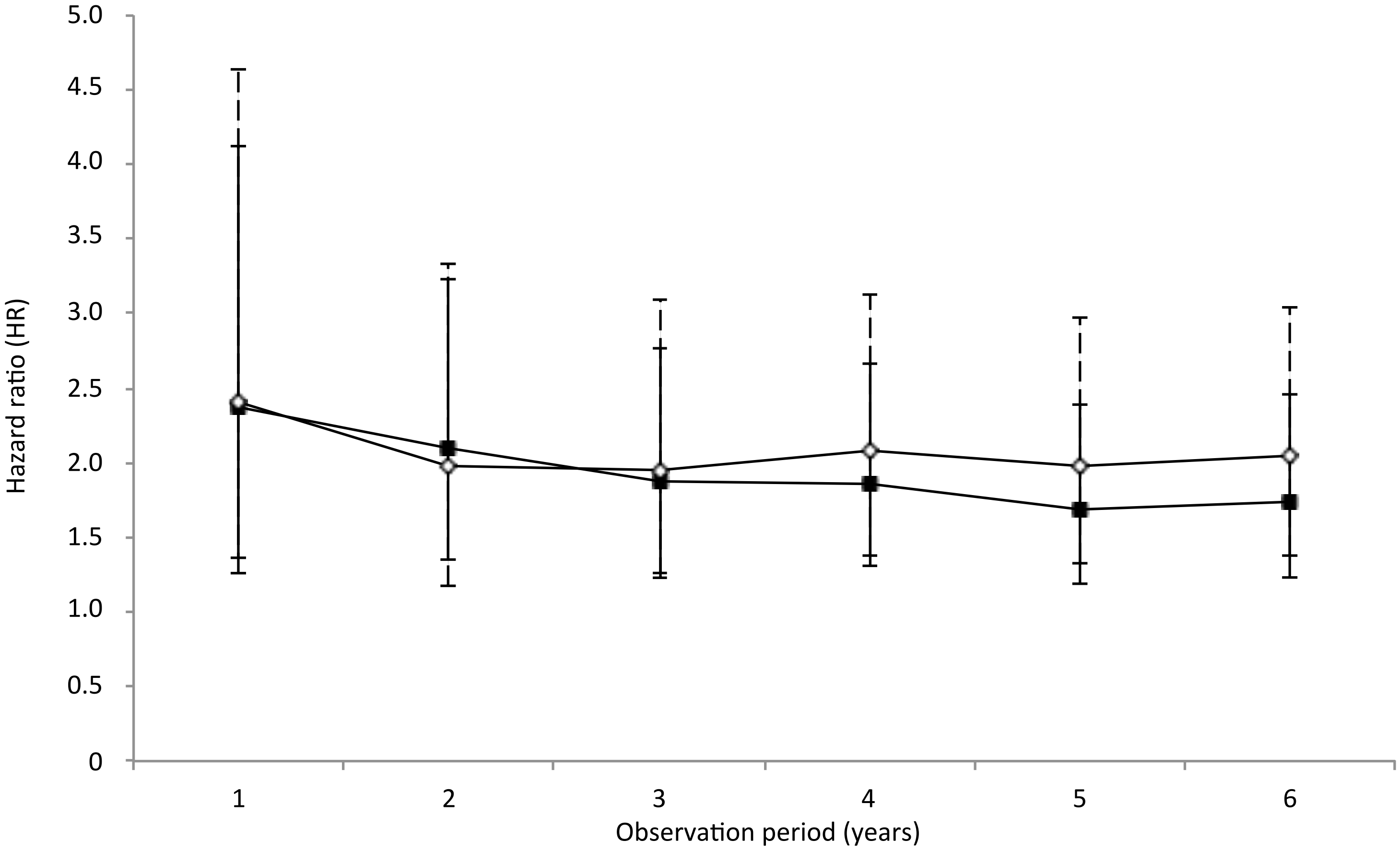

	1	2	3	4	5	6
HR for mild anemia	2.37	2.09	1.87	1.86	1.69	1.74
HR for moderate to severe anemia	2.42	1.98	1.95	2.07	1.98	2.05

Supplementary Material Figure S1. Hazard ratios (HR) for patients with AMI and anemia for increasing observation periods
Reference: Non-anemia: Hemoglobin (Hb) concentration of ≥12 g/dL in women, Hb concentration of ≥13 g/dL in men.
Mild anemia: Hb concentration of 11 g/dL to <12 g/dL in women, Hb concentration of 11 g/dL to <13 g/dL in men.
Moderate to severe anemia: Moderate to severe anemia: Hb concentration of <11 g/dL in women and men.
95% Confidence intervals (CI) are represented by vertical lines above and below the HR estimates; 95% CI for mild anemia: dashed line; 95% CI for moderate to severe anemia: continuous line.
