# Supplementary material for: Association between admission anemia and long-term mortality in patients with acute myocardial infarction: results from the MONICA/KORA myocardial infarction registry
Source: BMC Cardiovasc Disord. 2018 Mar 9;18:50. doi: 10.1186/s12872-018-0785-5 (PMC5845173; doi:10.1186/s12872-018-0785-5)
Supplement: Supplementary file 2 — Table S1. Results of the sensitivity analysis (n = 2187). (DOCX 16 kb) [file 12872_2018_785_MOESM2_ESM.docx]

**Table S1.** Results of the sensitivity analysis (n=2,187)

|  | **Anemia**^a^ (n=338) | | | | | | **Non-anemia**^d^ (n=1,849) |
| --- | --- | --- | --- | --- | --- | --- | --- |
|  | **Total** | | **Mild anemia**^b^ (n=209) | | **Moderate to severe anemia**^c^ (n=129) | |  |
|  | HR [95% CI] | p Value | HR [95% CI] | p Value | HR [95% CI] | p Value | HR [95% CI] |
| Model 3^e^ | 3.08 [2.42-3.92] | <0.0001 | 2.46 [1.81-3.33] | <0.0001 | 4.19 [2.07-5.72] | <0.0001 | Ref. |
| All-cause mortality, n (%) | 108 (32.0) | | 55 (26.3) | | 53 (41.1) | | 188 (10.2) |

AMI, acute myocardial infarction; CI, confidence interval; HR, hazard ratio; Ref., reference category.

^a^ Anemia: Hemoglobin (Hb) concentration of <12 g/dL in women, Hb concentration of <13 g/dL in men.

^b^ Mild anemia: Hb concentration of 11 g/dL to <12 g/dL in women, Hb concentration of 11 g/dL to <13 g/dL in men.

^c^ Moderate to severe anemia: Hb concentration of <11 g/dL in men and women.

^d^ Non-anemia: Hb concentration of ≥12 g/dL in women, Hb concentration of ≥13 g/dL in men.

^e^ Model 3: Adjusted for age and sex including patients with missing information on any covariate except Hb concentration (n=176).
